# Follistatin and Systolic Function at Follow-Up in STEMI Patients: A Cardiac Magnetic Resonance Study

**DOI:** 10.3390/jcm15155777

**Published:** 2026-07-23

**Authors:** Jose Gavara, Tamara Molina-García, Elena de Dios, Nerea Perez-Solé, Victor Marcos-Garces, Hector Merenciano-Gonzalez, Amparo Ruiz-Saurí, Antoni Bayés-Genis, Julio Nuñez, Vicente Bodí, César Rios-Navarro

**Affiliations:** 1 Instituto de Investigación Sanitaria INCLIVA, 46010 Valencia, Spain; jose.gavara@outlook.es (J.G.); elenaddll@gmail.com (E.d.D.); neere_8@hotmail.com (N.P.-S.); marcos_vic@gva.es (V.M.-G.); hectormeren@gmail.com (H.M.-G.); amparo.ruiz-sauri@uv.es (A.R.-S.); yulnunez@gmail.com (J.N.); 2 Centro de Investigación Biomédica en Red—Cardiovascular (CIBER-CV), 28029 Madrid, Spain; abayesgenis@gmail.com; 3 Instituto del Corazón, Hospital Universitari Germans Trias i Pujol, 08916 Badalona, Spain; 4 Departamento de Medicina, Facultad de Medicina, Universitat de València, 46010 Valencia, Spain; tamara5mg1999@gmail.com; 5 Servicio de Cardiología, Hospital Clínico Universitario de Valencia, 46010 Valencia, Spain; 6 Departamento de Patología, Facultad de Medicina, Universitat de València, 46010 Valencia, Spain

**Keywords:** follistatin, adverse left ventricular remodeling, cardiac magnetic resonance, ST-segment elevation myocardial infarction

## Abstract

**Background/Objectives**: Early circulating biomarkers capable of identifying patients at risk for impaired recovery of left ventricular (LV) systolic function after ST-segment elevation myocardial infarction (STEMI) remain limited. This exploratory study aims to determine whether serum follistatin measured within the first 24 h after coronary revascularization is associated with long-term LV systolic function, as assessed by cardiac magnetic resonance (CMR). **Methods**: In this prospective study, 31 patients with reperfused STEMI underwent serial CMR at 1 week and 6 months. Follistatin levels were measured within 24 h after revascularization. Associations between follistatin and CMR-derived LV systolic function were assessed using univariate and multivariable linear regression analyses. **Results**: Patients were stratified by median follistatin level (438 pg/mL) into below median (*n* = 15) and above median (*n* = 16). At 1 week, CMR-derived LV function, volumes, and structural indices were similar between groups. By 6 months, however, patients with follistatin above the median had higher LV ejection fraction (LVEF) [62 ± 9% vs. 54 ± 9%; *p*-value = 0.018] and lower LV end-systolic volume index (28 ± 12 vs. 41 ± 18 mL/m^2^; *p*-value = 0.037). In univariate analysis, follistatin was associated with 6-month LVEF (β = 0.027 per pg/mL; *p*-value = 0.004). After multivariable adjustment, follistatin remained independently associated with 6-month LVEF (β = 0.024 per pg/mL; *p*-value < 0.001) and with relative improvement in LVEF from 1 week to 6 months (β = 0.044 per pg/mL; *p*-value < 0.001). **Conclusions**: In reperfused STEMI patients, higher early follistatin levels were independently associated with more favorable LV systolic function at 6 months. These hypothesis-generating findings suggest that follistatin may represent a potential biomarker of subsequent systolic recovery after STEMI, warranting further validation in larger prospective studies.

## 1. Introduction

Despite major advances in timely reperfusion and secondary prevention, ST-segment elevation myocardial infarction (STEMI) remains associated with an increased risk of subsequent heart failure and death [1,2]. A key determinant of these events is adverse left ventricular (LV) remodeling (ALVR), a maladaptive process characterized by progressive changes in LV size, geometry, and systolic performance after infarction. As post-infarction ALVR is closely linked to late clinical outcomes, early identification of patients at risk is of relevance in the current management of STEMI patients [3].

Cardiac magnetic resonance (CMR) is the gold-standard technique for assessing post-infarction myocardial injury and LV remodeling, enabling accurate quantification of LV volumes and function together with tissue characterization of infarct size and microvascular injury. However, CMR identifies remodeling only after structural changes have developed during follow-up and the procedure is not always available or cost-effective [4,5]. From a clinical standpoint, circulating biomarkers obtained soon after revascularization could provide complementary risk stratification at a timepoint when decisions regarding optimization of medical treatment and clinical management are still actionable. There is thus considerable interest in evaluating circulating biomarkers that can be obtained early after revascularization and that might identify patients at risk of developing ALVR during follow-up.

Follistatin is an endogenous activin-binding protein with potential relevance to myocardial injury and repair [6]. Experimental evidence suggests that the activin–follistatin axis is activated during myocardial ischemia–reperfusion and may modulate inflammatory and injury responses [7,8,9]. In clinical studies, circulating follistatin levels have been reported to be higher in patients with myocardial infarction than in those with stable coronary artery disease [10], whereas related evidence indicates that increased activin A is associated with worse echocardiography-derived LV remodeling after STEMI [11]. Together, these observations support a biologically plausible link between early follistatin release and the subsequent remodeling response, but this association has not been established using a CMR-defined remodeling endpoint in a STEMI scenario. Accordingly, this exploratory study sought to determine whether serum follistatin measured within the first 24 h after revascularization for STEMI is associated with systolic function at follow-up as assessed by CMR.

## 2. Materials and Methods

### 2.1. Study Population

This study conformed to the principles for use of human subjects outlined in the Declaration of Helsinki. The study protocol was approved by the local Research Ethics Committee, and written informed consent was obtained from all subjects.

Inclusion criteria were patients with a first STEMI as outlined in current definitions treated with primary coronary intervention within 12 h of chest pain onset, and who underwent CMR imaging 1 week and 6 months post-STEMI. We prospectively enrolled 38 consecutive patients with these characteristics discharged between July 2022 and December 2024.

**Figure 1:**
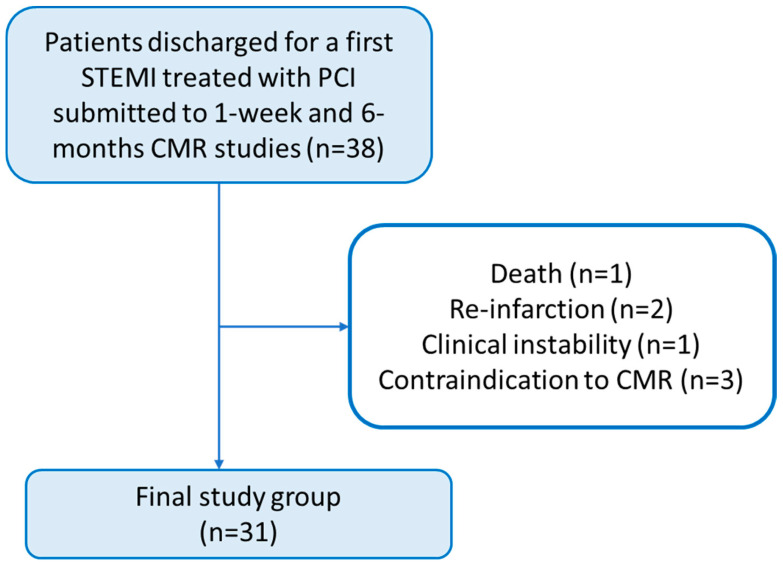
Patient enrolment flowchart. Flowchart of patient enrolment and reasons for exclusion, yielding the final cohort of 31 patients with paired CMR after STEMI. Abbreviations: CMR = cardiovascular magnetic resonance. PCI = percutaneous coronary intervention. STEMI = ST-segment elevation myocardial infarction.

Exclusion criteria were death (*n* = 1), re-infarction (*n* = 2), or clinical instability (*n* = 1) during the first six months post-discharge or any contraindication to CMR (*n* = 3). The final study group comprised 31 STEMI patients. The study patient flowchart is presented in Figure 1.

### 2.2. Biomarker Assays

Blood samples were isolated 24 ± 3 h after coronary revascularization, centrifuged at 2300 rpm for 15 min, and serum was immediately stored at −80°C until further analyses were performed. Serum follistatin concentrations were measured using the MILLIPLEX^®^ MAP Human Angiogenesis/Growth Factor Magnetic Bead Panel 1 assay (HAGP1MAG-12K, Merck Millipore, Darmstadt, Germany) according to the manufacturer’s instructions. Based on the kit datasheet, the minimum detectable concentration of follistatin is 11.1 pg/mL, the intra-assay coefficient of variation is <10% and the inter-assay coefficient of variation is <20%.

### 2.3. CMR Acquisition, Sequences and Quantification

Patients included in this study underwent two CMR studies (1.5 T unit, Magnetom Sonata; Siemens, Erlangen, Germany) at 7 [5,6,7,8] days (1-week CMR) and 196 [167–242] days (6-month CMR) after STEMI, in accordance with our laboratory protocol and current recommendations. All studies were performed and analyzed by two cardiologists specialized in CMR with 15 years of experience, using customized software (QMASS MR 6.1.5, Medis, Leiden, The Netherlands). CMR data were prospectively recorded and immediately included in the database.

In both studies, the following indices were calculated by manual planimetry of endocardial and epicardial borders in short-axis view cine images: LV ejection fraction (LVEF, %), LV end-diastolic volume index (LVEDVI, mL/m^2^), LV end-systolic volume index (LVESVI, mL/m^2^), and LV mass index (g/m^2^).

Further details regarding CMR acquisition, sequences and index quantification can be consulted elsewhere [4,12,13].

### 2.4. Endpoints and Follow-Up

The primary endpoints were the relationship between follistatin measured at 24 h after primary percutaneous coronary intervention and long-term LV remodeling, as reflected by 6-month LVEF and LVESVI.

### 2.5. Statistical Analysis

Data were tested for normal distribution using the Kolmogorov–Smirnov test. Continuous normally distributed data were expressed as the mean ± standard deviation of the mean and compared using paired and unpaired Student’s *t*-tests. Non-parametric data were expressed as the median with the interquartile range and compared using the Mann–Whitney U-test. Group percentages were compared using the Chi-square test or Fisher’s exact test where appropriate.

The relationship between follistatin (pg/mL) and 6-month LVEF (%), between follistatin (pg/mL) and 6-month LVESVI (mL/m^2^), between follistatin (pg/mL) and relative increase in LVEF from 1 week to 6 months (ΔLVEF, %), and between follistatin (pg/mL) and relative increase in LVESVI from 1 week to 6 months (ΔLVESVI, %) were analyzed using multivariate linear regression models in the entire study group. For the multivariate regression analyses, only baseline and CMR characteristics associated with follistatin in univariate linear regression analysis were chosen as covariates for adjustment (*p*-value < 0.05). Next, a reduced and parsimonious model was derived using backward stepwise selection. Collinearity of variables tested in the multivariate models was assessed using the tolerance statistic (excessive if <0.20) and the variance inflation factor (excessive if >5). Model performance and goodness-of-fit were evaluated using the coefficient of determination (R^2^), which is reported for all final multivariate models.

The covariates included in the final model were 1-week LVEF (%) and follistatin (pg/mL) for 6-month LVEF (%) and ΔLVEF (%), 1-week LVESVI (mL/m^2^) and follistatin (pg/mL) for 6-month LVESVI (mL/m^2^), and 1-week LVEDVI (mL/m^2^) and follistatin (pg/mL) for ΔLVESVI (%).

Median-based stratification was used for descriptive comparisons only, whereas all inferential analyses used follistatin as a continuous variable.

Statistical significance was set at a two-tailed *p*-value < 0.05. The SPSS statistical package (version 15.0, SPSS Inc., Chicago, IL, USA) and STATA (Version 9.0, StataCorp, College Station, TX, USA) were used throughout.

## 3. Results

A total of 31 patients were included (mean age 61 ± 11 years), of whom 25 (81%) were male. Anterior infarction was present in 17 (55%) patients, and three (10%) presented with Killip class ≥ II.

Serum follistatin concentrations measured 24 h after coronary revascularization showed a mean value of 464 ± 184 pg/mL. Patients were stratified by median follistatin level (438 pg/mL) into below-median (*n* = 15) and above-median (*n* = 16) groups. Between-group baseline clinical characteristics were similar, although anterior infarction was more frequent among patients with follistatin below the median (37.5% vs. 73.3%; *p*-value = 0.045). Medical treatment at discharge was similar in both groups.

Table 1 and Table 2 summarize baseline characteristics and CMR data at acute phase (1 week) and follow-up (6 months) stratified by follistatin levels.

### 3.1. Association of Follistatin with 1-Week and 6-Month CMR Parameters

At 1-week CMR study, LV function, ventricular volumes, and tissue-characterization parameters were similar in the two follistatin groups. LVEF was 54 ± 8% in patients with follistatin below the median and 57 ± 8% in those above the median (*p*-value = 0.275). Likewise, there were no between-group differences in LVEDVI (87 ± 29 vs. 81 ± 17 mL/m^2^; *p*-value = 0.492), LVESVI (41 ± 17 vs. 35 ± 11 mL/m^2^; *p*-value = 0.232), LV mass (86 ± 19 vs. 80 ± 9 g/m^2^; *p*-value = 0.281), edema burden (35 ± 14% vs. 32 ± 20% of LV mass; *p*-value = 0.574), microvascular obstruction (0.7 [0–4.2] vs. 0 [0–3.9]% of LV mass; *p*-value = 0.456), infarct size (21 ± 11% vs. 19 ± 14% of LV mass; *p*-value = 0.614), myocardial salvage index (39 ± 27% vs. 41 ± 25%; *p*-value = 0.863), or intramyocardial hemorrhage (0 [0–0] vs. 0 [0–0]; *p*-value = 0.762) (Table 2).

At 6 months, patients with follistatin above the median had higher LVEF than those with lower levels (62 ± 9% vs. 54 ± 9%; *p*-value = 0.018), while 6-month LVEDVI did not differ significantly between groups (74 ± 20 vs. 84 ± 24 mL/m^2^; *p*-value = 0.214). The higher-follistatin group also had lower 6-month LVESVI (28 ± 12 vs. 41 ± 18 mL/m^2^; *p*-value = 0.037), with a nonsignificant trend toward lower LV mass (68 ± 13 vs. 81 ± 23 g/m^2^; *p*-value = 0.070) and smaller infarct size (13 ± 10% vs. 19 ± 10% of LV mass; *p*-value = 0.139) (Table 2). Thus, associations with follistatin emerged primarily during follow-up rather than at the early post-infarction CMR study.

Longitudinal analyses were directionally concordant with these 6-month findings. ΔLVEF was numerically greater in patients with higher follistatin (7.0 ± 12.5% vs. 1.2 ± 11.5%), whereas ΔLVESVI was directionally more favorable (−12.1 ± 18.4% vs. 1.8 ± 29.4%). Although these between-group comparisons did not reach statistical significance when follistatin was analyzed dichotomously (*p*-value = 0.241 for ΔLVEF and *p*-value = 0.159 for ΔLVESVI), they were consistent with the continuous analyses and with the 6-month CMR profile (Table 2).

### 3.2. Predictors of 6-Month LVEF

In univariate analysis, lower 6-month LVEF was correlated with anterior infarction (β = −8.508; *p*-value = 0.014), lower 1-week LVEF (β = 0.778; *p*-value < 0.001), larger 1-week LV volumes, higher 1-week LV mass, greater edema burden, more extensive microvascular obstruction, larger infarct size, and lower follistatin levels (β = 0.027 per pg/mL; *p*-value = 0.004). Neither age, sex, cardiovascular risk factors, ischemic time, GRACE risk score, TIMI risk score, and multivessel disease, nor TIMI flow grades before or after primary coronary intervention were associated with 6-month LVEF in univariate testing (Table 3).

**Figure 2:**
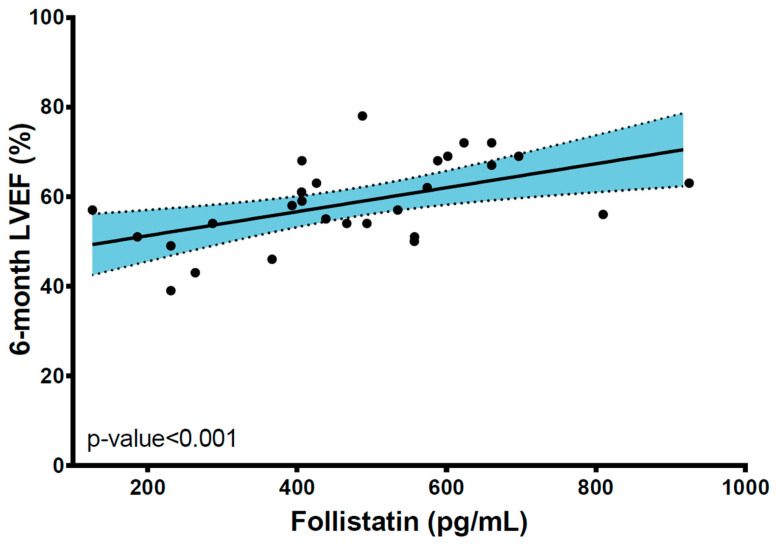
Association between early follistatin levels and 6-month left ventricular ejection fraction. The relationship between serum follistatin levels measured within 24 h after revascularization and LVEF at 6-month CMR follow-up in patients with reperfused STEMI obtained from the final multivariable linear regression models. Higher follistatin levels were associated with higher 6-month LVEF (*p*-value < 0.001). Abbreviations: CMR = cardiac magnetic resonance. LVEF = left ventricular ejection fraction. STEMI = ST-segment elevation myocardial infarction.

After multivariable adjustment, follistatin remained independently associated with 6-month LVEF (β = 0.024 per pg/mL; *p*-value < 0.001), together with 1-week LVEF (β = 0.647; *p*-value < 0.001). Notably, anterior infarction, microvascular obstruction, infarct size, LV mass, and other acute CMR markers were no longer significant once baseline systolic function and follistatin were accounted for. Expressed clinically, each 100-pg/mL increment in follistatin corresponded to an approximately 2.4 percentage-point higher 6-month LVEF. Figure 2 shows a direct association between follistatin levels and 6-month LVEF across the observed biomarker range.

**Figure 3:**
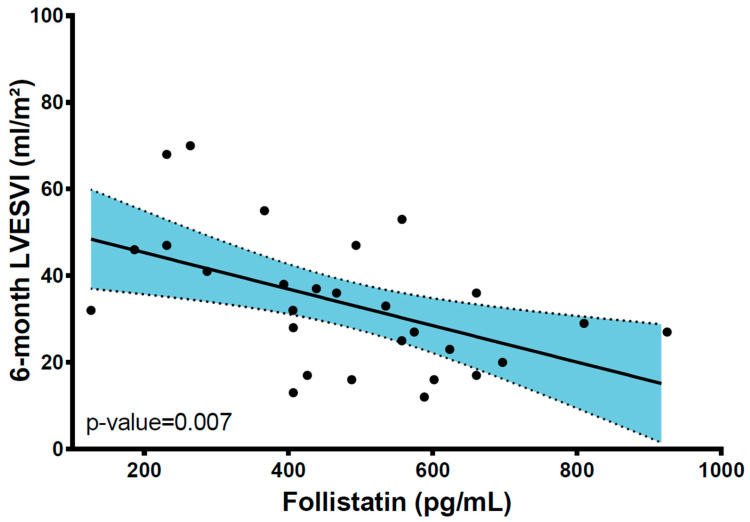
Association between early follistatin levels and 6-month left ventricular end-systolic volume index. The relationship between serum follistatin levels measured within 24 h after revascularization and LVESVI at 6-month CMR follow-up in patients with reperfused STEMI obtained from the final multivariable linear regression models. Higher follistatin levels were associated with lower 6-month LVESVI (*p*-value = 0.007). Abbreviations: CMR = cardiac magnetic resonance. LVESVI = left ventricular end-systolic volume index. STEMI = ST-segment elevation myocardial infarction.

A similar pattern was observed for longitudinal systolic recovery. For ΔLVEF, follistatin remained independently associated with greater LVEF improvement (β = 0.044 per pg/mL; *p*-value < 0.001), along with 1-week LVEF (β = −0.691; *p*-value = 0.003). Anterior infarction was no longer significant after adjustment (β = 0.046; *p*-value = 0.787). On a scaled basis, a 100-pg/mL higher follistatin level was correlated with an approximately 4.4% greater ΔLVEF. This continuous relationship can also be observed in Figure 3, where higher follistatin values tracked with greater gains in LVEF over time.

### 3.3. Predictors of 6-Month LV Volumes

Although the main indicator was long-term systolic function, volumetric analyses were also concordant. In univariate analysis, higher 6-month LVESVI was associated with anterior infarction (β = 12.710; *p*-value = 0.019), lower 1-week LVEF (β = −1.186; *p*-value < 0.001), larger 1-week LV end-diastolic and end-systolic volume indices, higher LV mass, greater microvascular obstruction, larger infarct size, and lower follistatin levels (β = −0.042 per pg/mL; *p*-value = 0.006). In the multivariable model, follistatin remained independently associated with 6-month LVESVI (β = −0.034 per pg/mL; *p*-value = 0.007), together with 1-week LVESVI (β = 0.617; *p*-value < 0.001) (Table 4).

**Figure 4:**
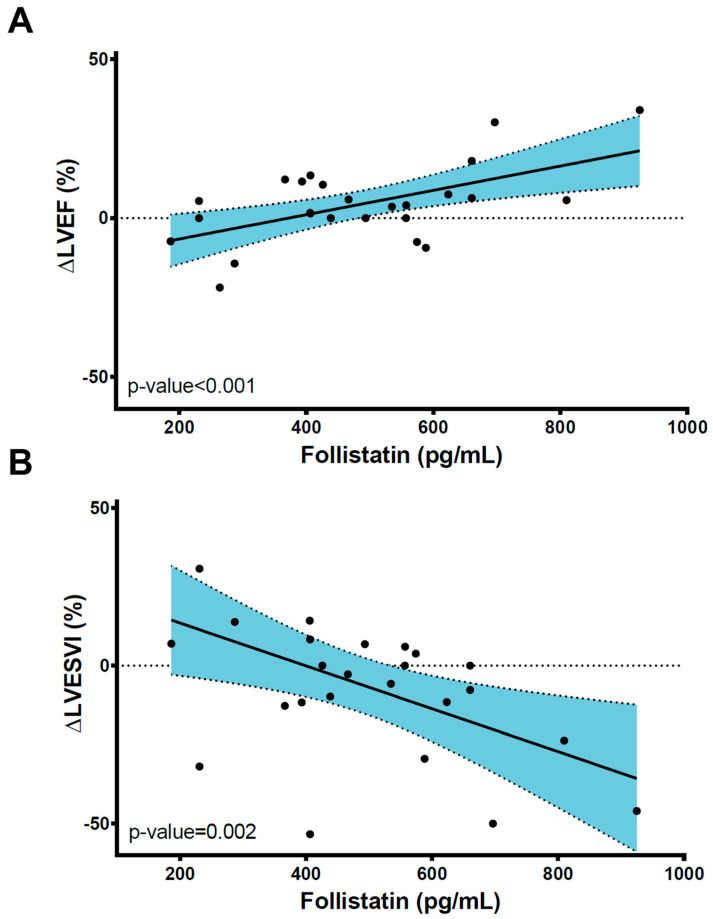
Association between early follistatin levels and longitudinal changes in left ventricular systolic function obtained from the final multivariable linear regression models. (**A**) Relationship between serum follistatin levels measured within 24 h after revascularization and relative change in LVEF from 1 week to 6 months. (**B**) Relationship between serum follistatin levels measured within 24 h after revascularization and relative change in LVESVI from 1 week to 6 months. Higher follistatin levels were associated with greater improvement in LVEF (*p*-value < 0.001) and greater reduction in LVESVI (*p*-value = 0.002) during follow-up. Abbreviations: LVEF = left ventricular ejection fraction. LVESVI = left ventricular end-systolic volume index.

For ΔLVESVI, follistatin also remained independently associated with a greater reduction over time (β = −0.080; *p*-value = 0.002), along with 1-week LVEDVI (β = −0.397; *p*-value = 0.030). Figure 4 showed an inverse correlation between follistatin and both 6-month LVESVI and ΔLVESVI. Overall, these volumetric findings were directionally aligned with the LVEF analyses, while the most consistent association involved 6-month systolic function.

## 4. Discussion

In this exploratory study, early follistatin levels measured within the first 24 h after reperfusion for STEMI patients were independently associated with long-term LV systolic function as assessed by CMR at 6 months. This correlation was more evident at follow-up than during acute phase, as early follistatin levels were not accompanied by major differences in LV function, volumes, or structural indices at 1 week. Higher follistatin levels were associated with patients showing a more favorable systolic recovery, correlated with higher 6-month LVEF and greater improvement in LVEF during follow-up. Taken together, these findings suggest that follistatin may serve as an early biomarker of subsequent LV functional recovery after STEMI, although further validation in larger prospective cohorts is needed.

### 4.1. Early Biomarkers and Long-Term Systolic Function After STEMI

In current STEMI care, early identification of patients at risk for impaired late LV systolic recovery remains a clinical need despite major advances in reperfusion therapy and secondary prevention [1,2,3]. Although CMR provides comprehensive characterization of post-infarction myocardial injury and ventricular remodeling, its value for risk stratification is inherently linked to imaging findings obtained after structural and functional changes have already developed [4,5]. In this line, advanced echocardiographic techniques, particularly speckle tracking echocardiography, may provide complementary information because myocardial deformation parameters, especially global longitudinal strain, can reveal subtle systolic dysfunction and have been linked to myocardial fibrosis and tissue abnormalities [14,15]. From a clinical perspective, an early circulating biomarker capable of identifying patients less likely to recover systolic function could be particularly useful to guide follow-up management after discharge. In fact, the integration of circulating biomarkers such as follistatin with speckle tracking echocardiography-derived deformation indices and CMR markers could support a more refined approach to identifying patients at risk of adverse ventricular remodeling or incomplete functional recovery after STEMI.

In the current exploratory study, our results suggest that the association between early follistatin and long-term systolic function was not mirrored by major differences in the acute CMR study. At 1 week, LVEF, LV volumes, edema burden, microvascular obstruction, infarct size, and myocardial salvage index were broadly similar across the two follistatin groups. Conversely, by 6 months, patients with higher follistatin levels showed higher LVEF and lower LVESVI, and follistatin remained independently associated with both 6-month LVEF and improvement in LVEF during follow-up after adjustment for anterior infarction and baseline CMR parameters. This pattern suggests that follistatin may capture biological processes more closely related to subsequent myocardial healing and functional recovery than to the initial severity of ischemic injury itself.

These results should also be interpreted in the context of prior studies showing that early circulating biomarkers after STEMI may identify the subsequent trajectory of LV systolic recovery, although no single biomarker has yet emerged as clearly dominant in this setting. Higher initial neprilysin levels, for example, have been associated with subsequent improvement in LVEF at follow-up [16]. In contrast, higher levels of soluble suppression of tumorigenicity 2 measured 24 h after reperfusion have been associated with lower follow-up LVEF, higher LV end-systolic volume on serial CMR and echocardiographic assessments [17,18]. Similarly, higher admission macrophage migration inhibitory factor levels have been linked to poorer recovery of echocardiography-derived cardiac function and worse long-term outcomes after STEMI [19], whereas higher early chitinase-3-like protein 1 levels have shown a negative correlation with CMR-LVEF recovery at 6 months [20]. Against this background, our findings extend the field by suggesting that higher early follistatin levels are also associated with a more favorable long-term systolic trajectory after reperfused STEMI.

### 4.2. Mechanistic Considerations of Follistatin and Post-MI Repair

Although further experimental and clinical experiments will be needed, the biological plausibility of our results could be supported by previous data involving the activin A-follistatin axis. Follistatin is an extracellular binding protein that neutralizes activin A, and imbalance of the activin A-follistatin system has been linked to tissue repair, fibrosis, and cardiomyocyte apoptosis in post-infarction heart failure models [21]. Although human data directly involving follistatin after myocardial infarction remain limited, circulating follistatin and activin A have been reported to be higher in patients with myocardial infarction than in those with stable coronary artery disease, suggesting early activation of this pathway after acute ischemic injury [10]. In the same signaling axis, activin A measured on day 2 in 278 patients with STEMI was associated with lower LVEF and higher LV end-diastolic volume index at 6 months and predicted all-cause death, linking early pathway activation with subsequent adverse ventricular evolution [11]. Experimental observations are directionally concordant. In a murine ischemia–reperfusion model, myocardial activin A and follistatin proteins increased early after injury whereas circulating follistatin decreased, and pretreatment with exogenous follistatin significantly reduced infarct extension in experimental models [7]. In rat post-infarction models, inhibition of activin A attenuated inflammatory signaling and LV dysfunction, whereas modulation of the activin A-follistatin balance with ramipril was associated with less fibrosis and collagen accumulation together with higher follistatin expression [22]. Additional *in vitro* and chronic post-infarction heart failure experiments suggest that excessive activin A signaling can promote endoplasmic reticulum stress-mediated cardiomyocyte apoptosis, with follistatin upregulated later as a potential counter-regulatory response [21]. Taken together, these observations are compatible with the hypothesis that higher early follistatin may reflect a more favorable adaptive response to post-infarction injury and repair. However, external validation in larger multicenter cohorts is required to determine whether follistatin provides incremental prognostic value beyond established clinical, biochemical, and CMR markers and whether it may contribute to early risk stratification after STEMI.

### 4.3. Implications for Risk Stratification After STEMI

The present findings may also have relevant clinical implications, which might be scrutinized in further experimental and clinical approaches. In routine practice after STEMI, patients at risk of persistent or incompletely recovered LV systolic dysfunction are often identified only later during follow-up, even though the degree of LVEF recovery after STEMI carries important prognostic information. In addition, repeat assessment of LV systolic function after the acute event may influence subsequent management, including optimization of heart failure-directed medical therapy [12,23]. In this context, our data suggest that early follistatin measurement may provide clinically useful information shortly after reperfusion. Although patients with higher and lower follistatin levels showed broadly similar CMR findings at one week, elevated follistatin levels were independently associated with higher LVEF at 6 months and with greater improvement in LVEF during follow-up.

These observations should be interpreted in the context of the multifactorial and complex pathophysiology of post-STEMI LV recovery. Infarct extension, microvascular injury, residual myocardial ischemia, inflammatory activation, and guideline-directed medical therapy have all been reported to influence adverse ventricular remodeling and systolic recovery after STEMI [3,24]. Concretely, infarct size and microvascular obstruction are considered major determinants of subsequent LV dilation and impaired systolic function after coronary revascularization and inflammatory activation actively participates in infarct healing and extracellular matrix remodeling [3,4,24,25,26]. Therefore, the relationship between follistatin and 6-month CMR-derived indices may partly reflect these underlying factors, and residual confounding cannot be excluded.

If our results were confirmed in larger studies, early follistatin assessment may ultimately contribute to risk stratification if validated in larger prospective cohorts. Indeed, our results should be viewed as hypothesis-generating, and follistatin should be considered a potential complement to, rather than a replacement for, established clinical and imaging-based (i.e., CMR and speckle tracking echocardiography) risk stratification.

### 4.4. Limitations of the Study

This study has several limitations. First, the cohort was modest in size (*n* = 31), which limits statistical power, reduces precision around effect estimates, and constrains the number of variables that can be reliably incorporated into multivariable models. This may partly explain why some between-group comparisons, particularly those related to longitudinal changes in LV parameters, did not reach statistical significance despite consistent directional trends. Second, no formal internal validation procedure was performed; therefore, the regression models should be interpreted as exploratory association models rather than validated prediction models. Third, follistatin was measured at a single early time point, and serial biomarker sampling was not available; accordingly, we could not characterize its temporal profile after STEMI or assess whether repeated measurements might provide incremental prognostic information. Fourth, our analyses were based on imaging surrogate endpoints, and the study was not powered to evaluate clinical outcomes such as heart failure hospitalization, ventricular arrhythmias, or mortality. Fifth, the absence of an external validation cohort and the potential selection bias introduced with the use of a serial CMR population may limit the generalizability of these findings. Therefore, our results should be considered hypothesis-generating and warrant confirmation in larger prospective studies. Finally, although periprocedural variables (i.e., access site and rate of cardiogenic shock) could influence patients’ prognosis [27], this information was not systematically registered in our database.

## 5. Conclusions

In this prospective cohort of patients with reperfused STEMI, early follistatin levels were independently associated with long-term LV systolic function at 6 months. These hypothesis-generating findings indicate an association between early follistatin levels and subsequent post-infarction LV remodeling. Further studies in larger prospective cohorts are needed to validate these observations, clarify the biological pathways underlying this association, and determine whether follistatin provides incremental clinical value beyond established clinical and CMR markers for follow-up decision-making after STEMI.

## Figures and Tables

**Table 1:** Baseline characteristics, angiographic indices, medical treatment at discharge, and biomarkers of the entire cohort and of patients with follistatin above or below the median.

	All Patients	Follistatin Below Median (<438 pg/mL)	Follistatin Above Median (>438 pg/mL)	*p*-Value
Number of patients	31	15	16	
Baseline characteristics				
Age (years)	61 ± 11	60 ± 12	62 ± 10	0.559
Male sex (%)	25 (81)	10 (67)	15 (94)	0.056
Diabetes mellitus (%)	6 (19)	3 (20)	3 (19)	0.930
Hypertension (%)	15 (48)	9 (60)	6 (38)	0.210
Dyslipidemia (%)	13 (42)	6 (40)	7 (44)	0.833
Smoker (%)	17 (55)	7 (47)	10 (63)	0.376
Killip class (%)				0.616
1	28 (90)	14 (93)	14 (88)	
2	2 (6)	1 (7)	1 (6)	
3	0 (0)	0 (0)	0 (0)	
4	1 (3)	0 (0)	1 (6)	
Time to reperfusion (min)	160 [99–387]	160 [98–397]	173 [99–436]	0.692
GRACE risk score	122 ± 26	121 ± 25	124 ± 26	0.728
TIMI risk score	2 [1–3]	2 [1–3]	3 [0–4]	0.545
Angiographic indices				
Anterior infarction (%)	17 (55)	11 (73)	6 (38)	0.045
Multivessel disease (%)	11 (35)	6 (40)	5 (31)	0.705
TIMI flow grade before PCI (%)				0.403
0	22 (71)	10 (67)	12 (75)	
1	6 (19)	4 (27)	2 (13)	
2	0 (0)	0 (0)	0 (0)	
3	1 (3)	0 (0)	1 (6)	
TIMI flow grade after PCI (%)				0.129
0	0 (0)	0 (0)	0 (0)	
1	0 (0)	0 (0)	0 (0)	
2	2 (6)	2 (13)	0 (0)	
3	27 (87)	12 (80)	15 (94)	
Medical treatment at discharge				
DAPT (%)	27 (87)	13 (87)	14 (87)	0.945
Statins (%)	29 (93)	14 (93)	15 (94)	0.962
ACEi/ARA/ARNi (%)	22 (71)	10 (67)	12 (75)	0.609
Beta-blockers (%)	31 (100)	15 (100)	16 (100)	1
Diuretics (%)	1 (3)	1 (7)	0 (0)	0.294
Anticoagulants (%)	12 (39)	7 (47)	5 (31)	0.379
Mineralocorticoid receptor antagonists (%)	2 (6)	2 (13)	0 (0)	0.131
Follistatin (pg/mL)	464 ± 184	314 ± 93	605 ± 127	<0.001

Abbreviations: ACEi = angiotensin converting enzyme inhibitors. ARA = angiotensin II receptor antagonists. ARNi = angiotensin receptor neprilysin inhibitors. DAPT = dual antiplatelet therapy. GRACE = Global Registry of Acute Coronary Events. PCI = percutaneous coronary intervention. TIMI = Thrombolysis in Myocardial Infarction.

**Table 2:** CMR characteristics of the entire cohort and of patients with follistatin above or below the median.

	All Patients	Follistatin Below Median (<438 pg/mL)	Follistatin Above Median (>438 pg/mL)	*p*-Value
Number of patients	31	15	16	
1-week CMR				
LVEF (%)	55 ± 8	54 ± 8	57 ± 8	0.275
LV end-diastolic volume index (mL/m^2^)	84 ± 23	87 ± 29	81 ± 17	0.492
LV end-systolic volume index (mL/m^2^)	38 ± 14	41 ± 17	35 ± 11	0.232
LV mass (g/m^2^)	83 ± 15	86 ± 19	80 ± 9	0.281
Edema (% of LV mass)	33 ± 17	35 ± 14	32 ± 20	0.574
MVO (% of LV mass)	0 [0–3.8]	0.7 [0–4.2]	0 [0–3.9]	0.456
Infarct size (% of LV mass)	20 ± 12	21 ± 11	19 ± 14	0.614
Myocardial salvage index (%)	40 ± 25	39 ± 27	41 ± 25	0.863
Intramyocardial hemorrhage (n of segments)	0 [0–0]	0 [0–0]	0 [0–0]	0.762
6-month CMR				
LVEF (%)	59 ± 9	54 ± 9	62 ± 9	0.018
LV end-diastolic volume index (mL/m^2^)	78 ± 22	84 ± 24	74 ± 20	0.214
LV end-systolic volume index (mL/m^2^)	34 ± 16	41 ± 18	28 ± 12	0.037
LV mass (g/m^2^)	73 ± 19	81 ± 23	68 ± 13	0.070
MVO (% of LV mass)	0 [0–0]	0 [0–0]	0 [0–0]	0.082
Infarct size (% of LV mass)	15 ± 10	19 ± 10	13 ± 10	0.139
Relative change from 1-week to 6-month				
ΔLVEF (%)	4.5 ± 12.2	1.2 ± 11.5	7.0 ± 12.5	0.241
ΔLVEDVI (%)	−2.7 ± 20.9	0.9 ± 23.5	−5.5 ± 19.2	0.460
ΔLVESVI (%)	−5.9 ± 24.4	1.8 ± 29.4	−12.1 ± 18.4	0.159

Abbreviations: CMR = cardiovascular magnetic resonance. LV = left ventricular. LVEF = left ventricular ejection fraction. MVO = microvascular obstruction. ΔLVEDVI = relative change in LV end-diastolic volume index from 1-week to 6-month CMR study. ΔLVEF = relative change in LVEF from 1-week to 6-month CMR study. ΔLVESVI = relative change in LV end-systolic volume index from 1-week to 6-month CMR study.

**Table 3:** Predictors of 6-month LVEF and ΔLVEF: Univariate and multivariate analysis.

	6-Month LVEF				ΔLVEF	
	Univariate Analysis		Multivariate Analysis		Multivariate Analysis	
	Unstandardized Beta Coefficients	*p*-Value	Unstandardized Beta Coefficients	*p*-Value	Unstandardized Beta Coefficients	*p*-Value
			R^2^ = 0.733; Constant = 10.260		R^2^ = 0.556; Constant = 21.200	
Age (years)	0.285 [−0.045–0.615]	0.088	–	–	–	–
Male sex (%)	−1.750 [−12.417–8.917]	0.739	–	–	–	–
Diabetes mellitus (%)	2.252 [−7.473–11.977]	0.638	–	–	–	–
Hypertension (%)	−3.214 [−10.583–4.155]	0.378	–	–	–	–
Dyslipidemia (%)	4.344 [−2.950–11.637]	0.232	–	–	–	–
Smoker (%)	5.396 [−1.844–12.636]	0.138	–	–	–	–
Killip class (%)	−1.163 [−7.371–5.046]	0.703	–	–	–	–
Time to reperfusion (min)	0.000 [−0.006–0.007]	0.886	–	–	–	–
GRACE risk score	−0.003 [−0.152–0.146]	0.967	–	–	–	–
TIMI risk score	0.660 [−1.220–2.540]	0.477	–	–	–	–
Anterior infarction (%)	−8.508 [−15.179–−1.837]	0.014	–	0.780	–	0.787
Multivessel disease (%)	−3.394 [−10.651–3.863]	0.345	–	–	–	–
TIMI flow grade before PCI (%)	−2.111 [−7.393–3.171]	0.418	–	–	–	–
TIMI flow grade after PCI (%)	−3.077 [−21.932–15.779]	0.740	–	–	–	–
DAPT (%)	3.154 [−5.873–12.181]	0.481	–	–	–	–
Statins (%)	5.034 [−7.010–17.079]	0.400	–	–	–	–
ACEi/ARA/ARNi (%)	0.937 [−6.958–8.832]	0.810	–	–	–	–
Beta-blockers (%)	11.935 [−7.995–31.866]	0.231	–	–	–	–
Diuretics (%)	−19.161 [−38.292–−0.030]	0.050	–	0.883	–	0.950
Anticoagulants (%)	2.680 [−4.485–9.845]	0.451	–	–	–	–
Mineralocorticoid receptor antagonists (%)	−18.200 [−31.216–−5.184]	0.008	–	0.883	–	0.950
1-week CMR						
LVEF (%)	0.778 [0.499–1.056]	<0.001	0.647 [0.404–0.890]	<0.001	−0.691 [−1.121–−0.261]	0.003
LV end-diastolic volume index (mL/m^2^)	−0.158 [−0.301–−0.015]	0.031	–	0.430	–	0.525
LV end-systolic volume index (mL/m^2^)	−0.397 [−0.588–−0.205]	<0.001	–	0.624	–	0.770
LV mass (g/m^2^)	−0.278 [−0.483–−0.073]	0.010	–	0.913	–	0.844
Edema (% of LV mass)	−0.227 [−0.421–−0.033]	0.023	–	0.199	–	0.297
MVO (% of LV mass)	−1.047 [−1.686–−0.408]	0.002	–	0.640	–	0.853
Infarct size (% of LV mass)	−0.413 [−0.622–−0.203]	<0.001	–	0.255	–	0.356
Myocardial salvage index (%)	0.114 [−0.009–0.238]	0.069	–	–	–	–
Intramyocardial hemorrhage (n of segments)	−1.865 [−4.548–0.818]	0.165	–	–	–	–
Follistatin (pg/mL)	0.027 [0.010–0.044]	0.004	0.024 [0.012–0.035]	<0.001	0.044 [0.024–0.064]	<0.001

Abbreviations: ACEi = angiotensin converting enzyme inhibitors. ARA = angiotensin II receptor antagonists. ARNi = angiotensin receptor neprilysin inhibitors. DAPT = dual antiplatelet therapy. CMR = cardiovascular magnetic resonance. GRACE = Global Registry of Acute Coronary Events. LV = left ventricular. LVEF = left ventricular ejection fraction. MVO = microvascular obstruction. PCI = percutaneous coronary intervention. TIMI = Thrombolysis in Myocardial Infarction. ΔLVEF = relative change in LVEF from 1-week to 6-month CMR study. Regression coefficients are shown with 95% confidence intervals. Model fit is reported using R^2^. Collinearity of variables tested in 6-month LVEF model was as follows: anterior infarction: tolerance statistic 0.75; variance inflation factor 1.33. Diuretics: tolerance statistic 0.84; variance inflation factor 1.19. Mineralocorticoid receptor antagonists: tolerance statistic 0.82; variance inflation factor 1.23. 1-week LVEF: tolerance statistic 0.96; variance inflation factor 1.04. LV end-diastolic volume index: tolerance statistic 0.84; variance inflation factor 1.19. LV end-systolic volume index: tolerance statistic 0.44; variance inflation factor 2.27. LV mass: tolerance statistic 0.76; variance inflation factor 1.31. Edema: tolerance statistic 0.85; variance inflation factor 1.17. MVO: tolerance statistic 0.79; variance inflation factor 1.26. Infarct size: tolerance statistic 0.68; variance inflation factor 1.46. Follistatin: tolerance statistic 0.96; variance inflation factor 1.04. Collinearity of variables tested in ΔLVEF model was as follows: anterior infarction: tolerance statistic 0.75; variance inflation factor 1.33. Diuretics: tolerance statistic 0.75; variance inflation factor 1.33. Mineralocorticoid receptor antagonists: tolerance statistic 0.75; variance inflation factor 1.33. 1-week LVEF: tolerance statistic 0.96; variance inflation factor 1.04. LV end-diastolic volume index: tolerance statistic 0.84; variance inflation factor 1.19. LV end-systolic volume index: tolerance statistic 0.44; variance inflation factor 2.27. LV mass: tolerance statistic 0.76; variance inflation factor 1.31. Edema: tolerance statistic 0.85; variance inflation factor 1.17. MVO: tolerance statistic 0.79; variance inflation factor 1.26. Infarct size: tolerance statistic 0.68; variance inflation factor 1.46. Follistatin: tolerance statistic 0.96; variance inflation factor 1.04.

**Table 4:** Predictors of 6-month LVESVI and ΔLVESVI: Univariate and multivariate analysis.

	6-Month LVESVI				ΔLVESVI	
	Univariate Analysis		Multivariate Analysis		Multivariate Analysis	
	Unstandardized Beta Coefficients	*p*-Value	Unstandardized Beta Coefficients	*p*-Value	Unstandardized Beta Coefficients	*p*-Value
			R^2^ = 0.631; Constant = 28.075		R^2^ = 0.406; Constant = 66.420	
Age (years)	−0.467 [−0.999–0.065]	0.083	–	–	–	–
Male sex (%)	8.081 [−7.422–23.585]	0.296	–	–	–	–
Diabetes mellitus (%)	−0.625 [−13.867–12.617]	0.924	–	–	–	–
Hypertension (%)	7.563 [−3.555–18.680]	0.175	–	–	–	–
Dyslipidemia (%)	−8.067 [−19.158–3.025]	0.148	–	–	–	–
Smoker (%)	−4.056 [−15.516–7.405]	0.475	–	–	–	–
Killip class (%)	3.529 [−5.960–13.017]	0.453	–	–	–	–
Time to reperfusion (min)	0.000 [−0.011–0.010]	0.924	–	–	–	–
GRACE risk score	−0.008 [−0.249–0.233]	0.947	–	–	–	–
TIMI risk score	−0.092 [−3.011–2.827]	0.949	–	–	–	–
Anterior infarction (%)	12.710 [2.241–23.179]	0.019	–	0.958	–	0.873
Multivessel disease (%)	−1.441 [−13.516–10.634]	0.809	–	–	–	–
TIMI flow grade before PCI (%)	1.005 [−8.136–10.146]	0.824	–	–	–	–
TIMI flow grade after PCI (%)	−6.586 [−29.994–16.821]	0.569	–	–	–	–
DAPT (%)	−0.551 [−15.242–14.140]	0.939	–	–	–	–
Statins (%)	4.287 [−15.322–23.897]	0.447	–	–	–	–
ACEi/ARA/ARNi (%)	−4.952 [−17.572–7.669]	0.429	–	–	–	–
Beta-blockers (%)	−17.323 [−49.643–14.998]	0.282	–	–	–	–
Diuretics (%)	33.839 [3.390–64.287]	0.031	–	0.089	–	0.426
Anticoagulants (%)	−6.198 [−17.644–5.247]	0.278	–	–	–	–
Mineralocorticoid receptor antagonists (%)	−26.967 [5.515–48.418]	0.015	–	0.089	–	0.426
1-week CMR						
LVEF (%)	−1.186 [−1.716–−0.656]	<0.001	–	0.212	–	0.975
LV end-diastolic volume index (mL/m^2^)	0.376 [0.155–0.596]	0.002	–	0.583	−0.397 [−0.754–−0.041]	0.030
LV end-systolic volume index (mL/m^2^)	0.752 [0.452–1.051]	<0.001	0.617 [0.322–0.911]	<0.001	–	0.616
LV mass (g/m^2^)	0.552 [0.221–0.883]	0.002	–	0.427	–	0.718
Edema (% of LV mass)	0.252 [−0.099–0.602]	0.152	–	–	–	–
MVO (% of LV mass)	1.525 [0.375–2.675]	0.011	–	0.311	–	0.675
Infarct size (% of LV mass)	0.555 [0.159–0.951]	0.008	–	0.521	–	0.742
Myocardial salvage index (%)	−0.136 [−0.354–0.083]	0.213	–	–	–	–
Intramyocardial hemorrhage (n of segments)	2.850 [−1.773–7.474]	0.216	–	–	–	–
Follistatin (pg/mL)	−0.042 [−0.071–−0.013]	0.006	−0.034 [−0.058–−0.010]	0.007	−0.080 [−0.126–−0.033]	0.002

Abbreviations: ACEi = angiotensin converting enzyme inhibitors. ARA = angiotensin II receptor antagonists. ARNi = angiotensin receptor neprilysin inhibitors. DAPT = dual antiplatelet therapy. CMR = cardiovascular magnetic resonance. GRACE = Global Registry of Acute Coronary Events. LV = left ventricle. LVESVI = left ventricular end-systolic volume index. MVO = microvascular obstruction. PCI = percutaneous coronary intervention. TIMI = Thrombolysis in Myocardial Infarction. ΔLVESVI = relative change in LV end-systolic volume index from 1-week to 6-month CMR study. Regression coefficients are shown with 95% confidence intervals. Model fit is reported using R^2^. Collinearity of variables tested in 6-month LVESVI model was as follows: anterior infarction: tolerance statistic 0.78; variance inflation factor 1.29. Diuretics: tolerance statistic 0.90; variance inflation factor 1.11. Mineralocorticoid receptor antagonists: tolerance statistic 0.90; variance inflation factor 1.11. 1-week LVEF: tolerance statistic 0.46; variance inflation factor 2.17. LV end-diastolic volume index: tolerance statistic 0.21; variance inflation factor 4.78. LV end-systolic volume index: tolerance statistic 0.92; variance inflation factor 1.08. LV mass: tolerance statistic 0.48; variance inflation factor 2.09. MVO: tolerance statistic 0.90; variance inflation factor 1.11. Infarct size: tolerance statistic 0.85; variance inflation factor 1.18. Follistatin: tolerance statistic 0.92; variance inflation factor 1.08. Collinearity of variables tested in ΔLVESVI model was as follows: anterior infarction: tolerance statistic 0.85; variance inflation factor 1.18. Diuretics: tolerance statistic 0.91; variance inflation factor 1.10. Mineralocorticoid receptor antagonists: tolerance statistic 0.91; variance inflation factor 1.10. 1-week LVEF: tolerance statistic 0.86; variance inflation factor 1.17. LV end-diastolic volume index: tolerance statistic 0.95; variance inflation factor 1.06. LV end-systolic volume index: tolerance statistic 0.21; variance inflation factor 4.9. LV mass: tolerance statistic 0.43; variance inflation factor 2.33. MVO: tolerance statistic 0.93; variance inflation factor 1.07. Infarct size: tolerance statistic 0.93; variance inflation factor 1.08. Follistatin: tolerance statistic 0.95; variance inflation factor 1.06.

## Data Availability

The data presented in this study are available on request from the corresponding authors. The data are not publicly available due to ethical restrictions.

## Abbreviations

The following abbreviations are used in this manuscript:

ALVR	Adverse left ventricular remodeling
CMR	Cardiac magnetic resonance
LV	Left ventricle
LVEDVI	Left ventricular end-diastolic volume index
LVEF	Left ventricular ejection fraction
LVESVI	Left ventricular end-systolic volume index
STEMI	ST-segment elevation myocardial infarction

## References

[1] Thrane P.G., Olesen K.K.W., Thim T., Gyldenkerne C., Hansen M.K., Stødkilde-Jørgensen N., Jakobsen L., Bødtker Mortensen M., Dalby Kristensen S., Maeng M. (2024). 10-year mortality after ST-segment elevation myocardial infarction compared to the general population. J. Am. Coll. Cardiol..

[2] Jenča D., Melenovský V., Stehlik J., Staněk V., Kettner J., Kautzner J., Adámková V., Wohlfahrt P. (2021). Heart failure after myocardial infarction: Incidence and predictors. ESC Heart Fail..

[3] Marcos-Garcés V., Bertolín-Boronat C., Merenciano-González H., Martínez Mas M.L., Climent Alberola J.I., López-Bueno L., Payá Rubio A., Pérez-Solé N., Ríos-Navarro C., de Dios E. (2025). Left ventricular remodeling after myocardial infarction-pathophysiology, diagnostic approach and management during cardiac rehabilitation. Int. J. Mol. Sci..

[4] Marcos-Garcés V., Perez N., Gavara J., Lopez-Lereu M.P., Monmeneu J.V., Rios-Navarro C., de Dios E., Merenciano-González H., Gabaldon-Pérez A., Cànoves J. (2022). Risk score for early risk prediction by cardiac magnetic resonance after acute myocardial infarction. Int. J. Cardiol..

[5] Gissler M.C., Antiochos P., Ge Y., Heydari B., Gräni C., Kwong R.Y. (2024). Cardiac magnetic resonance evaluation of LV remodeling post-myocardial infarction: Prognosis, monitoring and trial endpoints. JACC Cardiovasc. Imaging.

[6] Zhang L., Liu K., Han B., Xu Z., Gao X. (2018). The emerging role of follistatin under stresses and its implications in diseases. Gene.

[7] Chen Y., Rothnie C., Spring D., Verrier E., Venardos K., Kaye D., Phillips D.J., Hedger M.P., Smith J.A. (2014). Regulation and actions of activin A and follistatin in myocardial ischaemia-reperfusion injury. Cytokine.

[8] Wei Q., Liu H., Liu M., Yang C., Yang J., Liu Z., Yang P. (2016). Ramipril attenuates left ventricular remodeling by regulating the expression of activin A-follistatin in a rat model of heart failure. Sci. Rep..

[9] Wei Y., Walcott G., Nguyen T., Geng X., Guragain B., Zhang H., Green A., Rosa-Garrido M., Rogers J.M., Garry D.J. (2025). Follistatin from hiPSC-cardiomyocytes promotes myocyte proliferation in pigs with postinfarction LV remodeling. Circ. Res..

[10] Anastasilakis A.D., Koulaxis D., Kefala N., Polyzos S.A., Upadhyay J., Pagkalidou E., Economou F., Anastasilakis C.D., Mantzoros C.S. (2017). Circulating irisin levels are lower in patients with either stable coronary artery disease (CAD) or myocardial infarction (MI) versus healthy controls, whereas follistatin and activin A levels are higher and can discriminate MI from CAD with similar to CK-MB accuracy. Metabolism.

[11] Lin J.F., Hsu S.Y., Teng M.S., Wu S., Hsieh C.A., Jang S.J., Liu C.J., Huang H.L., Ko Y.L. (2016). Activin A predicts left ventricular remodeling and mortality in patients with ST-elevation myocardial infarction. Acta Cardiol. Sin..

[12] Gavara J., Marcos-Garces V., Lopez-Lereu M.P., Monmeneu J.V., Rios-Navarro C., de Dios E., Perez N., Merenciano H., Gabaldon A., Cànoves J. (2022). Magnetic resonance assessment of left ventricular ejection fraction at any time post-infarction for prediction of subsequent events in a large multicenter STEMI registry. J. Magn. Reson. Imaging.

[13] de Dios E., Rios-Navarro C., Pérez-Solé N., Gavara J., Marcos-Garcés V., Forteza M.J., Oltra R., Vila J.M., Chorro F.J., Bodi V. (2021). Overexpression of genes involved in lymphocyte activation and regulation are associated with reduced CRM-derived cardiac remodelling after STEMI. Int. Immunopharmacol..

[14] Lisi M., Cameli M., Mandoli G.E., Pastore M.C., Righini F.M., D’Ascenzi F., Focardi M., Rubboli A., Mondillo S., Henein M.Y. (2022). Detection of myocardial fibrosis by speckle-tracking echocardiography: From prediction to clinical applications. Heart Fail. Rev..

[15] Sonaglioni A., Nicolosi G.L., Rigamonti E., Lombardo M., La Sala L. (2022). Molecular approaches and echocardiographic deformation imaging in detecting myocardial fibrosis. Int. J. Mol. Sci..

[16] Legallois D., Macquaire C., Hodzic A., Allouche S., El Khouakhi I., Manrique A., Milliez P., Saloux E., Beygui F. (2020). Serum neprilysin levels are associated with myocardial stunning after ST-elevation myocardial infarction. BMC Cardiovasc. Disord..

[17] Miñana G., Núñez J., Bayés-Genís A., Revuelta-López E., Ríos-Navarro C., Núñez E., Chorro F.J., López-Lereu M.P., Monmeneu J.V., Lupón J. (2018). ST2 and left ventricular remodeling after ST-segment elevation myocardial infarction: A cardiac magnetic resonance study. Int. J. Cardiol..

[18] Kim M., Lee D.I., Lee J.H., Kim S.M., Lee S.Y., Hwang K.K., Kim D.W., Cho M.C., Bae J.W. (2021). Lack of prognostic significance for major adverse cardiac events of soluble suppression of tumorigenicity 2 levels in patients with ST-segment elevation myocardial infarction. Cardiol. J..

[19] Deng X.N., Wang X.Y., Yu H.Y., Chen S.M., Xu X.Y., Huai W., Liu G.H., Ma Q.B., Zhang Y.Y., Dart A.M. (2018). Admission macrophage migration inhibitory factor predicts long-term prognosis in patients with ST-elevation myocardial infarction. Eur. Heart J. Qual. Care Clin. Outcomes.

[20] Hedegaard A., Ripa R.S., Johansen J.S., Jørgensen E., Kastrup J. (2010). Plasma YKL-40 and recovery of left ventricular function after acute myocardial infarction. Scand. J. Clin. Lab. Investig..

[21] Liu M., Mao C., Li J., Han F., Yang P. (2017). Effects of the activin A-follistatin system on myocardial cell apoptosis through the endoplasmic reticulum stress pathway in heart failure. Int. J. Mol. Sci..

[22] Hu J., Wang X., Tang Y.H., Shan Y.G., Zou Q., Wang Z.Q., Huang C.X. (2018). Activin A inhibition attenuates sympathetic neural remodeling following myocardial infarction in rats. Mol. Med. Rep..

[23] Wu W.Y., Biery D.W., Singh A., Divakaran S., Berman A.N., Ayuba G., DeFilippis E.M., Nasir K., Januzzi J.L., Di Carli M.F. (2020). Recovery of left ventricular systolic function and clinical outcomes in young adults with myocardial infarction. J. Am. Coll. Cardiol..

[24] Frantz S., Hundertmark M.J., Schulz-Menger J., Bengel F.M., Bauersachs J. (2022). Left ventricular remodelling post-myocardial infarction: Pathophysiology, imaging, and novel therapies. Eur. Heart J..

[25] Troger F., Pamminger M., Poskaite P., Reindl M., Holzknecht M., Lechner I., Tiller C., von der Emde S., Kaser A., Oberhollenzer F. (2025). Clinical impact of persistent microvascular obstruction in CMR after reperfused STEMI. Circ. Cardiovasc. Imaging.

[26] Graesser C., Krefting J., Schwab M., Scheidhauer H., Novacek S., Armbruster N.L., Hinterdobler J., Rapelius S., Romer A., Rumpf P.M. (2026). Prolonged inflammation associates with greater infarct size and poor outcome after ST-segment elevation myocardial infarction. JACC Basic Transl. Sci..

[27] Tokarek T., Dziewierz A., Plens K., Rakowski T., Dudek D., Siudak Z. (2021). Radial approach reduces mortality in patients with ST-segment elevation myocardial infarction and cardiogenic shock. Pol. Arch. Intern. Med..

